# A blueprint for success in real-world evidence: “glocal” approach to building capabilities and generating impactful evidence

**DOI:** 10.3389/fphar.2023.1233617

**Published:** 2023-10-11

**Authors:** Kristoffer Larsen, Ryan N. Walton, Mohamed Elsayed, Andrey Ipatov, Faye Townsend-Holyoake, Sebastian F. A. Axelsson, Nacho Quinones, Rudiger Papsch, Jennifer Givens, Alexander Bedenkov, Michael Seewald

**Affiliations:** ^1^ AstraZeneca Medical, Global, Gothenburg, Sweden; ^2^ AstraZeneca Medical, Europe and Canada Region, Baar, Switzerland; ^3^ AstraZeneca Medical, International Region, Dubai, United Arab Emirates; ^4^ AstraZeneca Medical, International Region, Moscow, Russia; ^5^ IQVIA, EMEA Real World Solutions, Medical Evidence Practice, London, United Kingdom; ^6^ AstraZeneca Medical, Global, Gaithersburg, MD, United States; ^7^ AstraZeneca Medical, Global, Cambridge, United Kingdom; ^8^ AstraZeneca Medical, Global, Hamburg, Germany

**Keywords:** real-world evidence (RWE), real-world data (RWD), evidence, capabilities, medical affairs

## Abstract

The past decade has seen the increasing influence and relevance of real-world data (RWD) and real-world evidence (RWE) in healthcare decision making. The value added by RWD/RWE has prompted the pharmaceutical industry to develop high performing systems and practices to harness the power of evidence generated at the global level. However, this worldwide transformation provides outstanding opportunities to support capability building within local affiliates and to impact key country-level stakeholders through resulting evidence. Therefore, we present an Evidence Blueprint Initiative, which links the global and local (“glocal”) skills, and furthermore addresses the opportunities and gaps in evidence generation capabilities at the local level. Cross-functional experts were recruited at the local, regional, and global level to define best practices. A framework was developed to characterize the foundational expertise needed and to assess markets’ existing capabilities. Subsequently, targeted roadmaps were developed and implemented to build capabilities in specific areas within each affiliate. The impact from the Blueprint is encouraging, resulting in improved local evidence plans, established evidence teams, enhanced RWD use and strategic implementation of patient centric science in local affiliates. The success of the Blueprint resides in empowering affiliates to realise their local evidence generation ambitions and to match them to their local context. It strengthens and expands the ties between various parts of the organisation and the external environment while building fit-for-future evidence capabilities from local affiliates.

## 1 Highlights

• Real-world data and evidence is becoming increasingly impactful in healthcare and the pharmaceutical industry needs to have capabilities in place both globally and locally to harness these insights
• We detail an industry-leading Evidence Blueprint for how to expand evidence generation capabilities within local affiliates through collaboration between global and local functions
• The value of the Blueprint has been observed across a variety of local affiliates with positive impacts across healthcare stakeholders

## 2 Introduction

Real World Evidence (RWE) can be used to improve quality of care and clinical outcomes by providing a more comprehensive understanding of the effectiveness and safety of therapies (Szymański et al., 2023). As a result, RWE is now considered an additional important source for decision making across Healthcare, Health Authorities, Payers, and Pharmaceutical Industries (Food and Drug Administration, 2018; Facey et al., 2020; Rudrapatna and Butte, 2020).

Real World Data (RWD) refers to local healthcare information that is routinely collected in hospital registries, electronic healthcare records (EHR), patient and disease registries and health insurance databases (Dagenais et al., 2022) and is of utmost importance for any evidence generation initiative to have a local understanding of skills and capabilities to generate the appropriate evidence at the right time to the right stakeholder.

With an increased role of RWD/E in the drug development process, beginning from pre-clinical to post-approval phases (Khosla et al., 2018; Dagenais et al., 2022), RWD and RWE act complementary. RWE relies on RWD to generate insights; however, it is important to distinguish between the two concepts as different capabilities are required for each. The need for country specific evidence capabilities in RWD is driven by differences in data environments, partnership opportunities and external acceptability. Much of the health information needed to transform healthcare practice, including clinical trials, medical affairs (including evidence), market access, and corporate affairs, is available in electronic health records, registries, and other data sources. As local health data is rich and may be fragmented, the healthcare industry may play a crucial role to support RWE through data integration, analyses, and utilization. For example, several publications have emphasized the future strategic role of Medical Affairs, to catalyse local practice change and impact key stakeholders and decision makers on local levels [ (Rudrapatna and Butte, 2020; Khosla et al., 2018; McKinsey, 2019; Dagenais et al., 2022; Their, 2022)]. However, a ‘glocal’ approach to evidence generation capabilities from an end-to-end perspective still needs to be explored and evaluated.

In order to address local challenges, we present a modular framework for how to ‘glocally’ address opportunities and support local affiliates in building their integrated end-to-end evidence generation capabilities, positively impacting healthcare. This framework is designed for markets of all sizes and maturity levels in evidence generation, considering varied data environment and opportunities. Additionally, it factors in existing best practices and potential barriers at the local level. The modular framework has been leveraged across AstraZeneca affiliates to build fit-for-purpose evidence capabilities through comprehensive local self-assessment and the development of country-specific roadmaps for capability building. This initiative could therefore be an opportunity for other organizations striving to further establish the industry as a collaborative partner to positively impact healthcare through enhanced RWE generation capabilities and utilization of local RWD.

## 3 Approach

**FIGURE 1:**
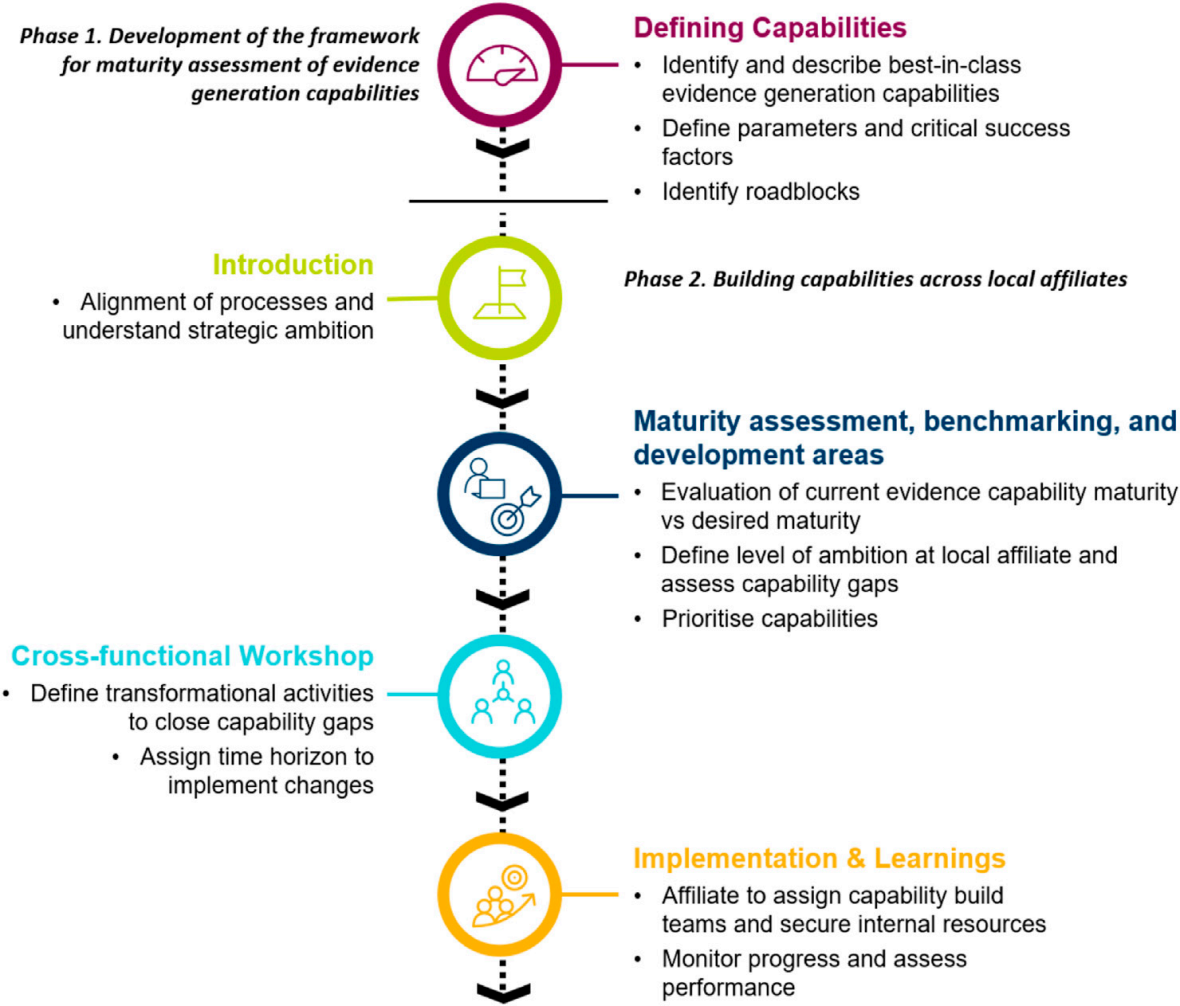
Overview of the processes of how the Evidence “Blueprint” capabilities framework was created and implemented to build evidence generation capabilities within local affiliates.

The Evidence Blueprint initiative was split into two phases: a framework development phase, and a subsequent framework implementation phase (see Figure 1 for an overview). The initial phase was to develop a modular framework that defines different levels of maturity of evidence generation capabilities. This “Blueprint” capability framework was subsequently launched as a tool for self-assessment among several voluntarily participating local affiliates. Based on the outcomes of the self-assessment, affiliates set their own benchmarks and aligned on the core areas for development. The assessment was followed by a workshop with the goal of creating a country-specific roadmap for capability building.

### 3.1 Development of the framework to allow assessment of evidence generation capabilities

**FIGURE 2:**
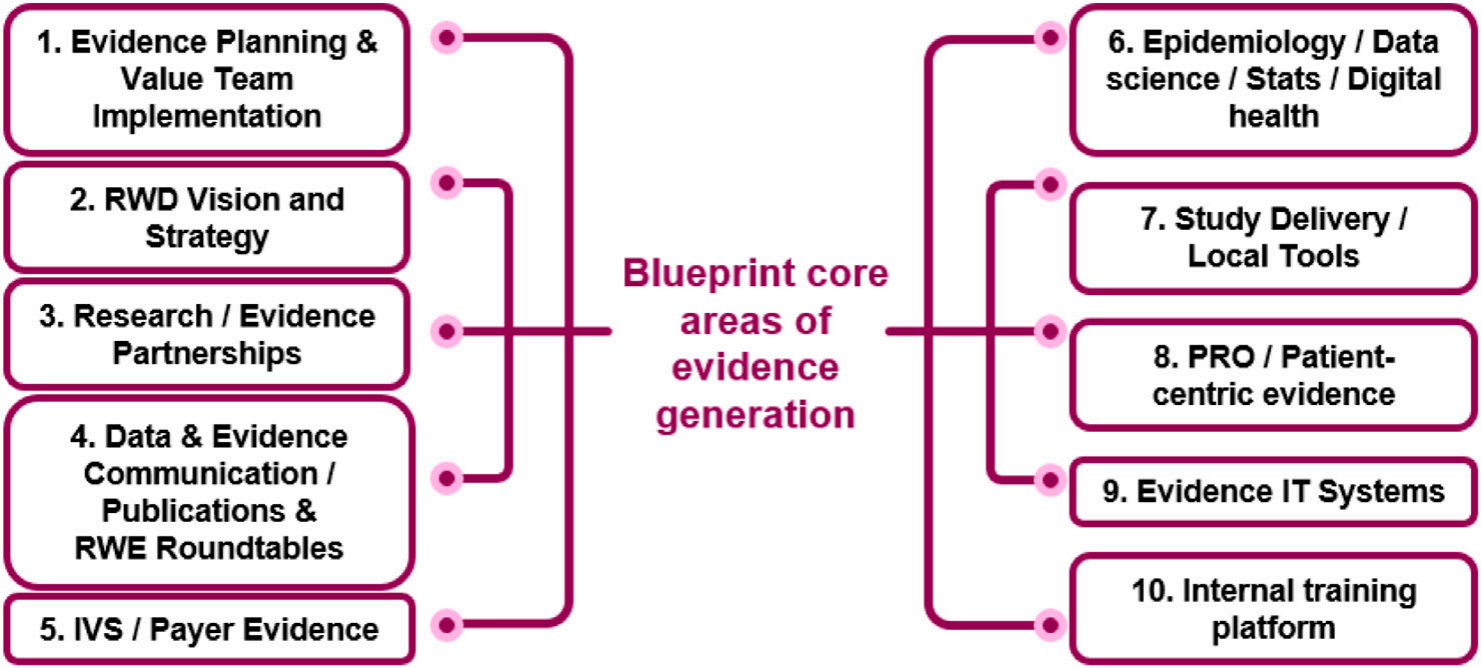
The Blueprint core-areas of evidence generation. The framework defines different levels of maturity (from emerging to leading) for each of the 10 areas. IVS innovative value strategies, PRO patient reported outcomes, *RWD* Real World Data.

The Evidence Blueprint capability framework was developed to enable best-in-class end-to-end evidence generation within the local affiliates of AstraZeneca. The framework is modular across different core areas of evidence generation, see Figure 2. The core areas were selected to cover all aspects of evidence generation, ranging from planning, strategy and vision, stakeholder specific evidence generation (i.e., payers and patients), dissemination, and evidence generation know-how. Each core area was developed independently and defined across four levels of maturity, ranging from emerging to leading.

The framework sets a benchmark for the foundational capabilities required to be a “leader” in evidence generation. A range of local, regional, and global AstraZeneca subject matter experts (SMEs) were engaged to catalogue best practices and define the various levels of maturity across the different core areas of evidence generation. As the framework was setup to develop internal ways of working, no external experts were engaged during initial development. The framework is comprehensive and considers key processes, tools and documents, people, Key Performance Indicators (KPIs) and stakeholders/communications. As each core area was developed independently, there was a subsequent centrally coordinated review to harmonize the defined levels of maturity for each area to ensure a high level of continuity across the Blueprint framework.

### 3.2 Implementation of the framework to build capabilities across local affiliates

The Blueprint forms the basis of the capability assessments across local affiliates, whereby an evaluation of current and desired maturity is evaluated, and an evidence capability roadmap is created to provide short-, mid- and long-term goals, actions, and critical success factors.

#### 3.2.1 Introduction

The Blueprint Initiative is *for* local affiliates and driven *by* local affiliates, however, the process of implementing the framework is supported by a core Blueprint team from AstraZeneca global Medical Affairs, responsible for defining the step approach and alignment on expectations (i.e., prioritized capabilities linked to strategic ambition of the local market). This is done jointly between the core team and key representatives of the local affiliate, most commonly the Medical Director and Evidence Generation Lead.

#### 3.2.2 Maturity assessment, benchmarking, and development areas

The self-assessment of evidence capability maturity is performed by a cross-functional team from the local affiliates. Typical functions that participate in the assessment are Country Medical Director, Medical Evidence Leads, Medical Advisors/Therapeutic Area Leads, Market Access, and a Commercial representation. However, this may differ depending on country set up. Each core capability is assessed based on current *versus* desired maturity level with regards to key processes, tools and documents, people, KPIs, and stakeholders/communications, Table 1 demonstrates the maturity framework for one of the modules. Following the assessment, local affiliates set their own benchmarks and align on the core capability areas for development. Areas are typically selected based on a balance between where the largest disparity between current and desired maturity exists, and to help develop areas that are expected to become important but currently have limited ongoing focus or investment.

**TABLE 1:** Mature assessment framework for RWD Vision and Strategy (core area 2). Evidence generation capabilities of a local affiliate can be assessed by a using a survey to understand the current and desired maturity for each core area. Each of the capability descriptors, for each core area is assessed in relationship to the maturities Emerging, Evolved, Advanced and Leading. AZ AstraZeneca, HTA health technology assessment, KPI Key Performance Indicator, RWD Real World Data, TA Therapy Area.

Capability descriptor (*RWD vision and strategy*)	Emerging	Evolved	Advanced	Leading
Processes	• No established processes in place for RWD strategy, quality assessment, access, or use • No mechanism to avoid duplicate licensing or avoid licensing of poor-quality data vs other local affiliates or globally available datasets	• Proactive landscaping of local data sources, new partners selected and validated, but not across all TAs and brands	• Data sources proactively and routinely identified based on TA and brand priorities, across all TAs and functions	• Taking a nationally leading role in shaping and driving country-wide strategies for health data collection, integration, governance, and stewardship • Create new or expand existing data sources across all TAs, e.g., by linking or by collaboration with data owners or vendors
		• Robust processes and framework in place to determine feasibility of research based on a given data source (prior to contracting)	• Quality assured, flexible framework delivering timely access to and analysis of RWD	
		• RWD strategy established across some TAs, as needed for key priorities, e.g., product launches	• Academic partnerships or commercial platforms leveraged as necessary when data cannot be directly accessed or licensed	
		• Some collaboration with internal stakeholders on RWD	• Consistent cross-functional collaboration on both routine deliverables as well as innovation	
Tools and Documents	• Limited awareness and use of tools and documents, reactive and *ad hoc* use of systems, tools and documents	• Tools and documents for identifying and evaluating RWD sources are established in select projects but not consistently across TAs and functions	• Robust tools and documents for identifying and evaluating RWD sources are established across all TAs and functions, aligned to established global toolboxes	• Tools, Documents, technologies, academic and vendor partnerships are exemplars and are considered industry leading and shared as best practices across other local affiliates and global
		• RWD priorities and strategy visibly documented across local affiliate stakeholders, in some not all TAs	• Seamless process working on new data licenses and partnerships with legal and procurement involved	
			• RWD priorities and long-term strategy established in all TAs	
**People/Role descriptions**	• Limited experience and focused on essential activities related to working with RWD	• Desired roles are all filled, and there are clear accountabilities for the team	• Team continues to evolve as new competencies are identified and brought into the team	• Team is seen as an exemplar in the region and AZ globally, and models for other local affiliates/regions
	• Experience dispersed across local affiliate, I.e., no cross-functional working group on RWD	• Active consideration of new competencies needed to address innovation in RWD, including digital sources	• Team structures are following global best practice	• Internal team operates at par with external thought-leaders, has a clear grasp and vision for local evolution of data landscape and tapping into it effectively
		• Continuing training and development of the team, includes potential cross functional secondments, assignments, or role shadowing	• Clear accountabilities between different functions that work with data	
**KPIs**	• KPIs identified and an initial assessment made of which can be adopted in-market	• KPIs are fully established in-market, and are consistently used to measure success in the use, evaluation, and integration of RWD	• Beyond measurement, KPIs are used to proactively identify areas of improvement in how the team uses RWD	• As team exceeds performance measured by existing KPIs, new performance measures are developed and placed, so that there is continued improvement
**Stakeholders & Communication**	• Reacting to project, RWD and analytical requests from internal stakeholders and delivering fit-for-purpose secondary studies	• Increased proactive scouting of new opportunities for RWD partnerships, new stakeholders evaluated and increased collaborations with data holders bringing diverse data sets	• Regular communication across all internal stakeholders, across all TAs and functions	• There is regular, proactive, 2-way communication between all stakeholders and partners, with the view to collaborate on new RWD sources, while enhancing existing RWD sources
		• Invited to RWD focused external roundtables or panel discussions to drive policy on use of RWD for routine HTA decisions	• Increasing external credibility and reliability such that AZ is sought as a partner to engage in health research • Collaborates actively with regulatory and reimbursement stakeholders to engage in discussions on RWD use for HTA or regulatory decision-making	• Proactively anticipating data needs (1-3 years ahead) aligned to business priorities and stakeholder needs and building stronger external relationships • Drives policy discussions on use of RWD for HTA and regulatory decision-making

#### 3.2.3 Cross-functional workshop

Following this assessment, a workshop is hosted for the local cross-functional team to align on strategies and jointly develop an evidence capability roadmap. The generated roadmap outlines the short-, mid- and long-term goals, actions, and critical success factors to enable the local affiliate to reach their desired maturity for the selected capability areas. The discussions and roadmap creation is driven by the local affiliate, with moderation provided by the global Blueprint core team. The global core team supports the process by sharing learnings from previous workshops; however, the direction and decisions are made by the local affiliate.

#### 3.2.4 Implementation and learnings

An outcome of the workshop is for the local affiliate to appoint a designated change lead that oversees the execution of the roadmap. The successes, learnings and roadblocks are shared with the global team in a follow-up call (approximately 6, 12 and 18 months after the workshop) but also informally with other local affiliates, contributing to the ever-growing knowledge sharing network that has been established as a result of the Blueprint Initiative.

## 4 Outcomes

The Blueprint Initiative has proven to be a hugely valuable approach that has enhanced the evidence generation capabilities across local affiliates. As a result of embarking on the Blueprint Initiative, local affiliates gain an in-depth understanding of their current capabilities by identifying areas of strength, and areas where there is room to improve to become leaders in evidence generation. An actionable plan to reach the desired capability level is developed as local affiliates define their own objectives and evidence generation ambitions in a cross-functional group. In this section we will present an overview of the rollout and highlight specific impacts of the initiative. Overall, the Blueprint has proven highly beneficial in understanding the level of intrinsic skills and capabilities necessary to generate the appropriate evidence at the right time to the right stakeholder.

### 4.1 Rollout of the initiative

Since 2021, ten local affiliates across Europe, North America, and Asia-Pacific have joined the Blueprint Initiative. The first ten countries had varying degrees of evidence generation capability maturity, broad geographical spread, and represented a variety of healthcare system archetypes. The local affiliates also represented different types of data infrastructure, highlighting differences in data collected across the markets.

Following the completion of the initiative by the first wave of 10 local affiliates, other markets have started to proactively express an interest to understand how they can also implement the Blueprint Initiative. Through the AstraZeneca company network, the impacts of the initiative have been circulated, creating a strong appetite among other affiliates for taking part in this evidence generation-empowering initiative, highlighting the importance of ‘glocal’ initiatives.

### 4.2 Impact

**FIGURE 3:**
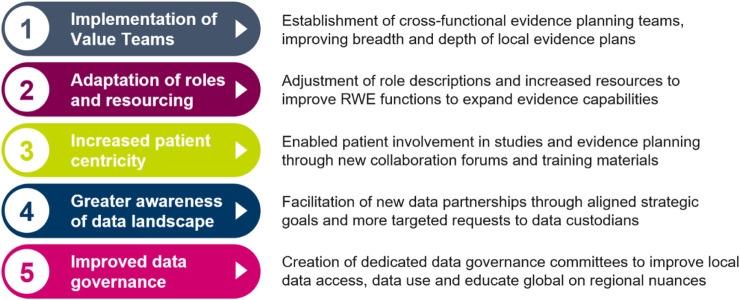
Five key impacts of the Blueprint that have contributed to improved evidence generation capabilities for local affiliates. RWE Real World Evidence.

**FIGURE 4:**
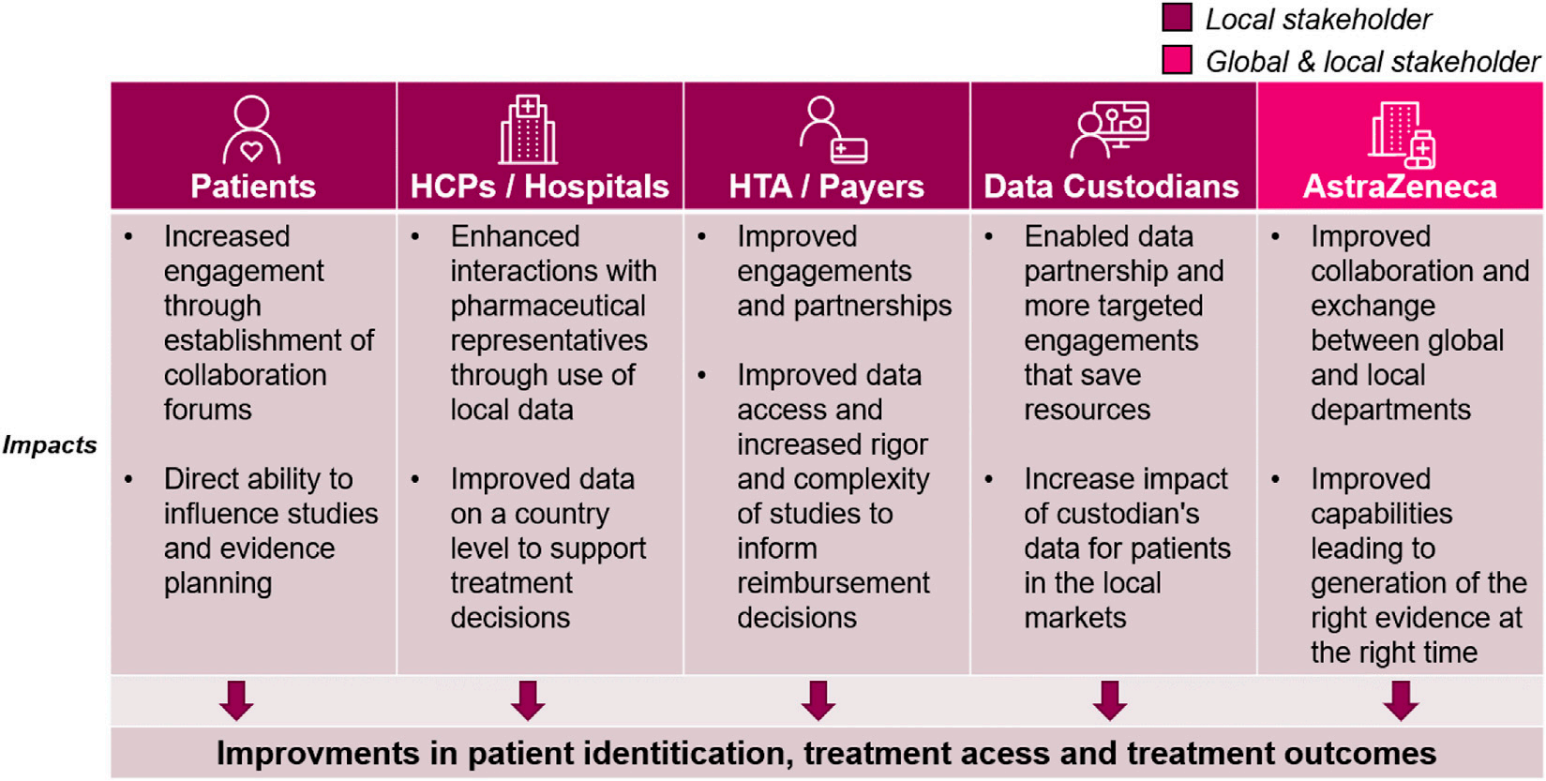
The impact of the Blueprint on healthcare stakeholders. While the blueprint initiative improves internal capabilities, the positive impacts can be seen across the local healthcare ecosystem. Each column outlines the impacts for the relevant stakeholders: Patients, Healthcare Providers (HCPs)/Hospitals at large, health technology assessment (HTA)/Payers, Data custodians (any stakeholder that owns and manages healthcare data for secondary use), and AstraZeneca global and local medical affairs*.*

Since the launch of the Evidence Blueprint in 2021, there have been vast and important improvements in local evidence capabilities in participating markets. The key improvements to capabilities are summarised in Figure 3, while the impact this has on healthcare stakeholders are summarised in Figure 4. Across local affiliates in both Europe and Asia-Pacific, one of the most significant impacts of the Blueprint Initiative is the establishment of strategic, cross-functional evidence planning teams. These specific Value Teams (VTs) target local evidence needs and opportunities for external impact with a cross-functionally aligned approach, for example, field communications, reimbursement dossiers, guideline development, and policy shaping. A local affiliate, representing one of the biggest four markets in the EU, have implemented therapeutic area VTs to ensure that dedicated cross-functional teams work towards the same strategic evidence goals for each specific disease area. This included engagement with the local senior management team to drive leadership support, research funding and team buy-in. Overall, the VTs have propelled a huge step change in both breadth and depth of evidence plans across local affiliates, with an observed increase in the complexity and rigor of study designs, including with use of more advanced analytical tools. Downstream and over time, this will bolster opportunities to drive external impact through resulting RWE data and publications, which can be leveraged for HCP interactions, reimbursement processes and other external engagement.

The Blueprint has also prompted local affiliates to adjust their evidence resourcing and role descriptions, improving local capacity and expertise. In a medium-sized central European market, the Blueprint played a key role in reigniting a collective belief in the importance of RWE and potential for the future, which led to increased RWE resource allocation, with specific expertise added in biostatistics and dedicated therapeutic area roles. In both previously mentioned markets, regular cross functional RWE sessions have been introduced, enabling better communication and more visibility within the therapeutic areas. This has led to better evidence strategy and evidence generation as an outcome.

Transforming patient care has taken centre-stage in catalysing RWD, and the Blueprint Initiative has played a pivotal role in embedding patient centricity in local evidence generation. Across local affiliates, there has been an emphasis on establishing collaboration forums with local patient societies and patient advocacy experts. In a North American market, for example, dedicated training materials are being created in collaboration with internal AstraZeneca Patient Engagement experts to educate local stakeholder on how best to engage with Patient Advocacy Groups (PAGs) on evidence generation. As a result, PAGS can be increasingly involved in more aspects of the study process, such as reviewing of study protocols. For example, a top European local affiliate is learning first-hand how to take their feedback into consideration throughout the lifecycle of a study by inviting third party patient association representatives to evidence planning workshops. Overall, this has led to new qualitative studies being developed across markets to capture patient experience.

The Blueprint has also prompted greater awareness of the regional data landscape for affiliates, establishing new data partnerships in key disease areas based on the evidence gaps identified by the VTs. Local affiliates, such as the top 4 EU market mentioned in the previous paragraph, conducted a review of their data supply in the context of local evidence needs. Findings were leveraged to continue support of data platforms and databases consortiums to integrate data for local evidence generation studies. This has resulted in targeted external partnerships to broaden access to data across primary, secondary, and tertiary cases. Across markets, processes have been established to better link evidence needs and data supply, e.g., implementation of local data sources to medical field staff to enhance customer interactivities, with a more frequent review of evidence needs via cross functional forums. Additionally, with increased patient centricity and improved data partnerships, local affiliates have expressed a wish to further evolve the use of data with patients. The evolved uses of data include engaging with patients/PAGs by leveraging the evidence to educate patients on how to advocate for themselves using evidence, e.g., in interactions with physicians.

Data governance is often concurrently managed by various functions, e.g., medical, commercial, and epidemiology. Following the Blueprint Initiative, some local affiliates have established dedicated data governance committees to ensure better visibility and safety of the data being accessed by the various cross functional partners. The data governance committees have allowed for reduced duplication of efforts and improved accountability in whom should be leading the analysis of specific data assets and also improved already robust data safety processes. This has resulted in more targeted external strategic engagement and made it possible to improve the enterprise strategy for data access and use across the affiliate. In addition to improvements locally, the data governance committee helps educate global functions on regional difference in data access.

## 5 Challenges and learnings

While the overall success of the Blueprint has been confirmed in the engagement with local affiliates throughout the programme, a few common challenges were observed across markets. There was not always complete alignment on the overall long-term evidence strategy. It was therefore important to ensure that the Blueprint roadmaps were developed to complement and advance the overall strategic objectives of the cross-functional evidence generation teams.

Additionally, a challenge that was encountered for some local affiliates was limited previous opportunities to benchmark their current capabilities within evidence generation. To pre-empt this challenge, we ensured that the framework was designed in collaboration with representative local affiliate SMEs to ensure that the defined levels of maturity would resonate across markets and reflect the full range of evidence capability levels. This means that the framework can be used to build capabilities from emerging markets to markets with strong capabilities in place already.

Additionally, through discussions during the benchmarking process, we were able to ensure that the local affiliates had a unified perspective on their current capability maturity, to successfully plan for building the capability regardless of current maturity.

As the role of global medical affairs is only acting as a catalyst in supporting evidence generation capability growth, a challenge has been the heterogenous nature of local affiliates and their needs. The team has had to adapt the timeline of the process and the level of support required depending on the maturity and skillsets of the local affiliates. The key to successful adaptation by the global team is to understand the current state of the local affiliate and also to pre-empt the needs and support required. An example of this is from one of the largest Asian markets, where additional support was provided from the global team in generating buy-in across the local affiliate, e.g., through support with material tailoring and messaging development, or jointly presenting the opportunities and success stories for the leadership team at the local affiliate.

We acknowledge that a limitation of the current approach is that the data-points in the framework and in the outcomes are subjective. Nonetheless, we believe the framework is completely fit-for-purpose to align subjective data with local strategies for evidence capability building and not to compare across local affiliates.

## 6 Future direction for building evidence capabilities across markets through the blueprint

The Blueprint is considered a living and evolving programme. As local affiliates share their experiences and feedback, the process and framework are updated and extended. The updates are related to both fine-tuning of the existing modules as well as extending the framework to additional modules to ensure it mirrors the ever-changing evidence generation landscape. As a future direction, we are considering the value of inviting external experts/stakeholders to comment on the framework to make sure capabilities developed are fit for their needs, e.g., PAGs to provide input on PRO/Patient centric evidence module. At the request of local affiliates and in line with broader strategic aims, a new module is planned to support the use of evidence in customer interactions in the field to drive medical practice change improving healthcare. Feedback received has also helped in fuelling other initiatives, such as a new global online repository that is under development. The support for the repository partly comes from the Blueprint and will be a “one stop shop” for materials that support evidence capabilities across the company. Additional initiatives that have emerged out of the Blueprint is an informal “matching” of local affiliates of different capability maturity, that can help complement and support each other with evidence generation initiatives.

After implementing the framework across 10 local affiliates, with many more underway, the strength in this initiative lies in “glocal” collaboration. With feedback and updates from a wide group of stakeholders, a positive feedback loop has been created that further strengthens the framework, improving the usefulness of the tool in building evidence capabilities across countries. We are certain that similar approaches within other pharma companies would be successful, but we also believe that similar approaches could be leveraged in non-profit settings with a global and local organisation to improve the impact of evidence generated.

In this publication, we have outlined a framework for how global healthcare companies can work to develop local affiliates in building their integrated end-to-end evidence generation capabilities, as well as the impact this framework has had. Since evidence and data should underpin all impactful medical activities, the ability to generate local impact on healthcare providers, patients, payers, and regulators is expected to be a crucial strategic driver for medical affairs now and into the future. The Blueprint framework offers a structured, fit-for-purpose approach for local capability building in the increasingly important field of RWE generation.

## References

[B1] Dagenais S. Russo L. Madsen A. Webster J. Becnel L. (2022). Use of real‐world evidence to drive drug development strategy and inform clinical trial design. Clin. Pharmacol. Ther. 111 (1), 77–89. 10.1002/cpt.2480 34839524 PMC9299990

[B2] Facey K. M. Rannanheimo P. Batchelor L. Borchardt M. de Cock J. (2020). Real-world evidence to support payer/HTA decisions about highly innovative technologies in the EU—actions for stakeholders. Int. J. Technol. Assess. Health Care 36 (4), 459–468. 10.1017/S026646232000063X 32878663

[B3] Food and Drug Administration (2018). Framework for FDA’s real world evidence program. Available at: https://www.fda.gov/media/120060/download (Accessed February 21, 2023).

[B4] Khosla S. White R. Medina J. Ouwens M. Emmas C. Koder T. (2018). Real world evidence (RWE) – A disruptive innovation or the quiet evolution of medical evidence generation? F1000Research 7, 111. 10.12688/f1000research.13585.2 30026923 PMC6039945

[B5] McKinsey (2019). A vision for medical affairs in 2025. Available at: https://www.mckinsey.com/∼/media/mckinsey/industries/life%20sciences/our%20insights/a%20vision%20for%20medical%20affairs%20in%202025/a-vision-for-medical-affairs-in-2025.pdf (Accessed February 21, 2023).

[B6] Rudrapatna V. A. Butte A. J. (2020). Opportunities and challenges in using real-world data for health care. J. Clin. Investigation 130 (2), 565–574. 10.1172/JCI129197 PMC6994109 32011317

[B7] Szymański P. Weidinger F. Lordereau-Richard I. Himmelmann A. Arca M. Chaves J. (2023). Real world evidence: Perspectives from a European society of cardiology cardiovascular round table with contribution from the European medicines agency. Eur. Heart J. - Qual. Care Clin. Outcomes 9 (2), 109–118. 10.1093/ehjqcco/qcad009 36746430

[B8] Their IQVIA. (2022). Finest hour: Medical affairs in a disrupted world. Available at: https://www.iqvia.com/-/media/iqvia/pdfs/library/white-papers/their-finest-hour-medical-affairs-in-a-disrupted-world.pdf (Accessed February 21, 2023).

